# Early Celiac Plexus Block in Metastatic Pancreatic Cancer: A Case Report

**DOI:** 10.1002/ccr3.71255

**Published:** 2025-10-10

**Authors:** Brian Fardman, Christina Sheedy, Joshua Raymond

**Affiliations:** ^1^ Rowan‐Virtua School of Osteopathic Medicine Stratford New Jersey USA; ^2^ Family Medicine Department Rutgers Robert Wood Johnson, CentraState Medical Center Freehold New Jersey USA

## Abstract

Both Early and Delayed Celiac Plexus Blocks (CPBs) have been used in pancreatic cancer. Early CPB is completed when the criteria for CPB are initially met, whereas Delayed CPB is implemented only after recalcitrant pain persists. We describe the benefit of early CPB in pancreatic cancer management.

## 1 Introduction

The celiac plexus is a visceral nerve bundle located behind the pancreas. It overlies the anterolateral surface of the aorta and innervates many visceral organs, including the pancreas. The plexus has been established as a targeted area for therapy in pancreatic cancer [1]. Celiac Plexus Block (CPB) is a minimally invasive procedure that has been shown to reduce pain and improve the quality of life in patients. The implementation of Early versus Delayed CPB has also been studied; the use of an Early CPB has been shown to decrease overall narcotic consumption and reduce adverse effects due to analgesic use when compared to Delayed CPB [2]. Early CPB is defined as an intervention initiated promptly once a patient meets the criteria, with abdominal pain being the most common indicator [3, 4]. Delayed CPB takes a wait‐and‐see approach and is only implemented when the patient develops persistent refractory pain or has intolerable adverse effects from analgesics [4]. This article presents a case in which early CPB was performed in a 73‐year‐old female who had pancreatic adenocarcinoma with lung metastases. The patient presented to CentraState Medical Center (CSMC) and underwent the procedure prior to the onset of recalcitrant abdominal pain and the need for a long course of analgesics. Early interventional treatment has been shown to be extremely beneficial in cancer patients [5]. This case report adds to the current belief that intervention with CPB should be early in patients with pancreatic cancer and will benefit them more than a wait‐and‐see approach by decreasing adverse effects from analgesics, improving patient comfort, and allowing for better patient care.

## 2 Case History/Examination

A 73‐year‐old female presented to CentraState Medical Center (CSMC) with an abdomen that was non‐tender on palpation, although lab reports were abnormal. Her past medical history was significant for paroxysmal supraventricular tachycardia, hypertension, and hyperlipidemia. Home medications included Digoxin 125 mg daily and Losartan 50 mg daily. Simvastatin had previously been prescribed but was discontinued at the recommendation of her physician. She reported no relevant family medical history. The patient resides in St. Crioux and had presented to a local hospital approximately one month prior with generalized jaundice. Imaging at that time revealed a pancreatic mass, prompting her to seek further evaluation in the United States. At the time of presentation to CSMC, she reported mild fatigue, occasional nausea, and infrequent, mild abdominal pain. Imaging performed at CSMC revealed a mass located at the head of the pancreas. She denied chest pain, shortness of breath, nausea, vomiting, diarrhea, numbness, weakness, tingling, or dizziness. Physical examination demonstrated no acute distress but was notable for jaundice, scleral icterus, and hepatomegaly, with the liver edge palpable 1 cm below the right costal margin.

## 3 Differential Diagnosis, Investigation, and Treatment

After collecting the patient's symptoms and history, several differential diagnoses were considered. Pancreatic cancer was of primary concern due to the patient's presentation and previous imaging. Additionally, a pancreatic cystic neoplasm or benign cyst was on our differential due to the patient's symptoms. However, these lesions are typically asymptomatic and rarely cause jaundice, making them less likely. Because autoimmune pancreatitis can often mimic malignancy, this was also on our differential. Lastly, metastatic disease was included due to the likelihood of certain malignancies like renal cell carcinoma, breast carcinoma, or bronchial carcinoma spreading to the pancreas. Lab work was completed to help guide diagnosis and treatment (Table 1).

### 3.1 Labs on Presentation

**TABLE 1:** Patient's labs on presentation to CentraState Medical Center.

Value	Result	Normal range	Interpretation
Total bilirubin	22.2	0.2–1.2 mg/dL	High
Conjugated bilirubin	10.2	0–0.5 mg/dL	High
CA 19‐9	35,858	< 35 U/mL	Very high
Glucose	139	65–99 mg/dL	High
AST (SGOT)	198	5–34 U/L	High
ALT (SGPT)	192	0–55 U/L	High
Alkaline phosphatase	526	40–150 U/L	High
Lipase	437	8–78 U/L	High
Urine Bilirubin	2+	Negative	Positive
Urobilinogen	2	0.2–1.0 EU/dL	High
CEA	8.3	0–3 ng/mL	High
AFP	2.32	0–6 ng/mL	Normal
GGT	534	5–55 U/L	High

### 3.2 Imaging on Presentation

**FIGURE 1:**
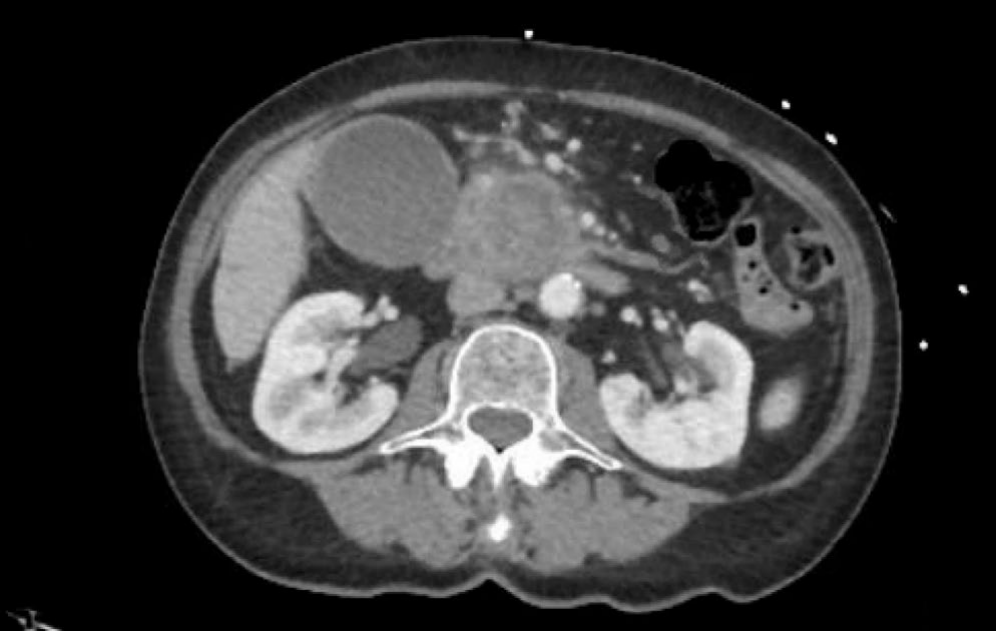
CT Abdomen showing pancreatic head mass with pancreatic ductal dilatation, consistent with pancreatic adenocarcinoma.

**FIGURE 2:**
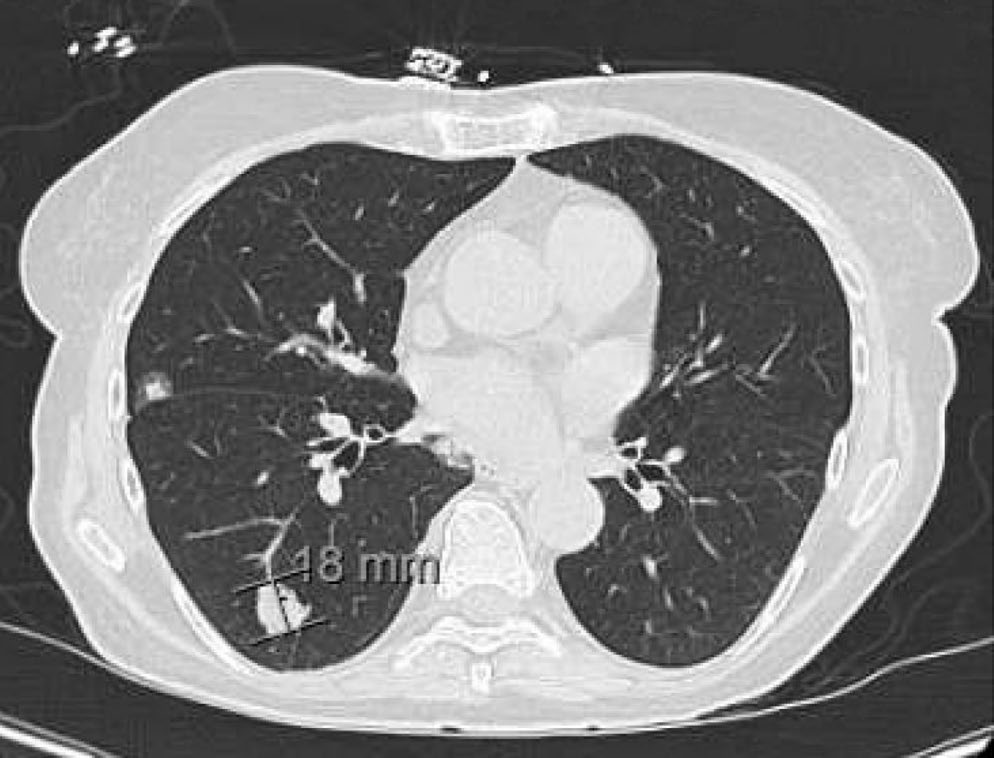
CT Chest showing pulmonary nodule suggestive of metastatic disease.

Computed Tomography (CT) of abdomen/pelvis with contrast showed multiple bilateral pulmonary nodules in the lung bases as well as a hypodense rounded lesion in the head of the pancreas with upstream dilatation of the pancreatic duct concerning for pancreatic adenocarcinoma (Figure 1). An ultrasound of the abdomen showed sludge in a distended gallbladder and a positive sonographic Murphy's sign. Intrahepatic biliary ducts were diffusely dilated and the common bile duct was markedly enlarged measuring 14 mm. The pancreatic head mass measured 4.1 × 4.1 cm and pancreatic ductal dilatation measured 6 mm. A CT scan of the chest showed bilateral pulmonary nodules and masses seen with bilateral lower lobe infiltrates (Figure 2). Percutaneous transhepatic cholangiogram demonstrated an obstructed and dilated intrahepatic biliary system with a stenotic common bile duct; a percutaneous biliary catheter was subsequently placed.

### 3.3 Procedures Throughout the Hospital Stay

Endoscopic Ultrasound‐guided Fine Needle Aspiration showed adenocarcinoma of the pancreatic head. A stomach biopsy produced oxyntic mucosa with preserved architecture; no intestinal metaplasia, dysplasia, or inflammation was noted. A lung biopsy confirmed a right lung nodule consistent with metastatic adenocarcinoma of the pancreas.

The preliminary diagnosis was thought to be a pancreatic tumor. This was prompted by the patient's presentation, imaging, and lab values. Further investigation confirmed this preliminary diagnosis, with biopsies revealing pancreatic adenocarcinoma with lung metastasis.

Treatment and management protocols were promptly discussed with the patient. A biliary drain and a duodenal stent were placed two weeks before the CPB procedure. Additionally, a chemotherapy port was inserted at this time, and the patient was informed that chemotherapy treatment could be anticipated once her bilirubin decreased to below 2 mg/dL. After these interventions were completed, the patient continued to have occasional nausea, decreased appetite, and mild transient abdominal pain. Interventional Radiology was then consulted for discussion of intervention with CPB. The Interventional Radiology team agreed that the CPB would be beneficial for the patient. The risks and benefits of early CPB were discussed with the patient. The patient decided to pursue the CPB, and the procedure was completed by the Interventional Radiology team. The patient tolerated the procedure well, and there were no complications.

## 4 Outcome and Follow‐Up

After the CPB was completed, the patient was then discharged to a subacute rehabilitation facility. She immediately endorsed relief of abdominal pain after the procedure. She has not needed any analgesics for abdominal pain one month post‐CPB. The patient reports being comfortable and has not had any abdominal complaints since the procedure. The patient will continue to be closely monitored for any abdominal discomfort, tenderness, or pain. Chemotherapy will begin in the near future.

## 5 Discussion

In summary, a 73‐year‐old female diagnosed with pancreatic cancer received a celiac plexus block early on in her course, prior to experiencing recalcitrant abdominal pain, to help prevent the onset of worsening symptoms. The patient tolerated the procedure well and had no complaints afterwards. The patient's final diagnosis was determined to be pancreatic adenocarcinoma with lung metastasis. This was supported by CT abdomen/pelvis, CT chest, chest XR, US abdomen, lung biopsy, and Endoscopic Ultrasound‐Guided Fine Needle Aspiration. No current evidence contradicted the diagnosis that was determined.

This case shows that Early Celiac Plexus Block helps improve quality of life, decreases the use of analgesics, and lessens the chances of adverse effects of narcotics in patients with pancreatic cancer. Up to 80% of patients with pancreatic cancer will experience pain during their course, so intervention should be proactive and early [6]. Early CPB will allow for better daily functioning and will improve patients' well‐being. Because the majority of patients experience pain, early intervention can be offered to help with the longitudinal treatment of pain. The conversation for CPB should be had with the patient whenever they initially meet criteria. A study comparing neurolytic sympathectomy before Step 2 of the World Health Organization (WHO) analgesic ladder with intervention after Step 3 demonstrated that early intervention led to a better response and reduced opioid consumption during the first 12 months of care [7]. This shows that waiting for a patient to need increased pain control potentially results in worsened outcomes. Step 1 on the WHO analgesic ladder is defined as mild pain that can be managed with NSAIDs or Acetaminophen. Step 2 is described as moderate pain that is managed with weak opioids or non‐opioid analgesics. Step 3 is defined as severe and persistent pain that requires potent opioids in addition to non‐opioid analgesics [8]. Intervening early with a CPB will allow for significantly decreased analgesic use and increased pain management control in patients with pancreatic cancer [9]. This case report adds to the literature that shows early intervention with Celiac Plexus Block will lead to better pain control, improved outcomes, and decreased side effects from analgesics in patients with pancreatic cancer.

## Author Contributions

**Brian Fardman:** investigation, resources, writing – original draft, writing – review and editing. **Christina Sheedy:** conceptualization, investigation, writing – original draft, writing – review and editing. **Joshua Raymond:** conceptualization, methodology, supervision, writing – review and editing.

## Consent

Written informed consent was obtained from the patient to publish this report in accordance with the journal's patient consent policy.

## Conflicts of Interest

The authors declare no conflicts of interest.

## Data Availability

All relevant data is included within the article, and no additional data are available.
